# Current challenges in the diagnosis of zearalenone toxicosis as illustrated by a field case of hyperestrogenism in suckling piglets

**DOI:** 10.1186/s40813-018-0095-4

**Published:** 2018-09-12

**Authors:** Isabel Hennig-Pauka, Franz-Josef Koch, Simone Schaumberger, Bettina Woechtl, Johannes Novak, Michael Sulyok, Veronika Nagl

**Affiliations:** 1 Field Station for Epidemiology, University of Veterinary Medicine Hannover, Foundation, Buescheler Straße 9, 49456 Bakum, Germany; 2 Tierarztpraxis im Holbeinring, Holbeinring 16, 35369 Gießen, Germany; 3 BIOMIN Holding GmbH, Erber Campus 1, 3131 Getzersdorf, Austria; 4 University Clinic for Swine, Department for Farm Animals and Veterinary Public Health, University of Veterinary Medicine Vienna, Veterinaerplatz 1, 1210 Vienna, Austria; 5 Functional Botanical Substances, Institute of Animal Nutrition and Functional Plant Compounds, Department for Farm Animals and Veterinary Public Health, University of Veterinary Medicine Vienna, Veterinaerplatz 1, 1210 Vienna, Austria; 6 Center for Analytical Chemistry, Department for Agrobiotechnology (IFA-Tulln), University of Natural Resources and Life Sciences, Vienna (BOKU), Konrad Lorenz Str. 20, 3430 Tulln, Austria; 7 BIOMIN Research Center, Technopark 1, 3430 Tulln, Austria

**Keywords:** Mycotoxins, Modified mycotoxins, Zearalenone, Metabolites, Hay, Splay leg, Gestating sow, Biomarker, Liquid chromatography, Tandem mass spectrometry

## Abstract

**Background:**

The mycotoxin zearalenone (ZEN) causes functional and morphological alterations in reproductive organs of pigs. In the field, diagnosis of ZEN-induced disorders is often challenging, as relevant feed lots are no longer available, or feed analysis results are not conclusive. Here, we report a field case of hyperestrogenism in newborn piglets. Surprisingly, more than 50 fungal metabolites were detected in hay pellets fed to gestating sows, including ZEN and its modified form zearalenone-14-sulfate (ZEN-14-S). Despite the broad contamination range in this unconventional feed component, a definite diagnosis of mycotoxicosis could not be achieved. In this context, current limitations regarding the confirmation of suspected cases of ZEN-induced disorders are discussed, covering both feed analysis and the biomarker approach.

**Case presentation:**

A piglet producer with 200 sows experienced a sudden increase in suckling piglet losses up to 30% by lower vitality and crushing. Predominant clinical signs were splay legs and signs of hyperestrogenism such as swollen and reddened vulvae in newborn piglets. The first differential diagnosis was ZEN mycotoxicosis although feed batches had not been changed for months with the exception of ground hay pellets, which had been included in the diet five months before. Analysis of hay pellets resulted in a sum value of ZEN and its modified forms of more than 1000 μg/kg, with ZEN-14-S alone accounting for 530 μg/kg. Considering the inclusion rate of 7% in the diet for gestating sows, the severe impact of the additional ZEN load due to the contaminated hay pellets seemed unrealistic but could not be completely excluded either. One month after hay pellets had been removed from the diet no further clinical signs were observed.

**Conclusions:**

Enrichment materials and other fibre sources can contain significant amounts of mycotoxins and should be therefore included in feed analysis. Adequate methods for broad spectrum mycotoxin determination, including modified mycotoxins, are important. As highlighted by this field case, there is a need to establish reliable biomarkers for ZEN exposure in pigs. Currently, available biomarkers do not allow a solid prediction of the ZEN intake of pigs under field conditions, which limits their application to experimental studies.

## Background

Mycotoxins are toxic secondary metabolites of molds, which lead to concentration dependent adverse health effects in pigs. In Northern and Central Europe, mycotoxins produced by species of genus *Fusarium*, such as *Fusarium graminearum* or *F. culmorum*, are most frequently detected in animal feeds [1]. Together with deoxynivalenol (DON), zearalenone (ZEN) is regarded as one of the most relevant *Fusarium* mycotoxins for pig production. ZEN is a frequent contaminant of maize, but can also occur in various other commodities, such as wheat, barley, or oats [2]. In a recent study, 88% of tested feed samples were tested positive for ZEN, with median and maximum concentrations of 20 μg/kg and 11192 μg/kg [3].

ZEN exhibits low acute toxicity but acts as full and partial agonist on estrogen receptors α and β, respectively [2]. As a consequence, ZEN exposure of pigs results in reproduction disorders. For example, mammary gland development is triggered in prepuberal gilts fed diets containing 1000–5000 μg/kg ZEN. In addition, vulvovaginitis, edema of vulva and rectal prolapses are observed [4, 5]. In mature sows, the effects of ZEN are highly dependent on feed concentration, exposure duration and phase of gestation, and range from anestrus to a decrease in live embryos, increase in dead-born piglets and abortions [6]. In newborn piglets of exposed sows, enlargement of external genitalia and a higher incidence of piglets with splay leg and trembling were recorded [5]. In addition, ZEN activates different receptors (most prominently the pregnane X receptor) involved in the regulation of cytochrome P450 isoforms in vitro, thus potentially affecting the phase I metabolism of various endo- and xenobiotics [7].

Pigs are especially susceptible to the effects of ZEN, which is attributed to the species-specific metabolism of this mycotoxin. After ingestion, ZEN is reduced to α- and β-zearalenol (α-ZEL and β-ZEL), mainly in intestinal or liver cells [8, 9]. Those phase I metabolites exert higher (α-ZEL) or lower (β-ZEL) biological activity than ZEN on estrogen receptors [10]. Hence, the rate of α-hydroxylation, which is comparably high in pigs [9], influences the toxicological potency of ZEN. Besides reduction, pathways of ZEN metabolism in animals comprise hydroxylation and glucuronidation, whereby major differences between species are observed [11]. Notably, ZEN undergoes substantial enterohepatic recirculation in pigs prior to its excretion via feces or urine [12].

In 2006, the European Commission introduced guidance levels for ZEN for compound feed. For pigs, recommended maximum values range from 100 μg/kg in compound feed for piglets and gilts to 250 μg/kg in compound feed for sows and fattening pigs [13]. However, the occurrence of modified mycotoxins is not taken into consideration in these regulations. Biologically modified mycotoxins result from modification of the mycotoxins´ chemical structures by plants, fungi or mammals [14]. For example, 17 different metabolites were detected in the model plant *Arabidopsis thaliana* after ZEN treatment, including zearalenone-14-*O*-β-glucoside [15]. Formation of zearalenone-14-sulfate (ZEN-14-S) was demonstrated in fungi [16, 17], and to a lesser extent also in plants [15]. So far, quantification of those mycotoxin conjugates is not implemented in routine analytical methods, although several studies indicated their widespread occurrence not only in feed, but also in food commodities [18]. ZEN and DON conjugates formed by plants or fungi seem to exhibit only limited intrinsic toxicity in mammals but pose a significant risk for animal health due to their hydrolysis in the digestive tract [19]. Thus, a substantial proportion of the parent toxin is liberated and adds up to the total mycotoxin burden.

Minimum welfare standards for pigs include the permanent access to enrichment objects and materials to enable explorative behavior [20, 21]. Appropriate enrichment material is deformable, destructible, ingestible, chewable and odorous [22]. These criteria are fulfilled by straw and hay, which are easily available in most geographical regions. Some reports about the mycotoxin load of grass exist, which was considered of major importance for ruminants [23]. On ryegrass, the symbiotic growth of the mold *Epichloë festucae* var. *lolii* led to the production of mycotoxins (ergovaline, lolitrem B). Recently, up to 77 fungal metabolites were detected in natural grasses (*Poaceae*) intended for grazing cattle [24], among them several mycotoxins such as ZEN (up to 2000 μg/kg), T-2 toxin, HT-2 toxin, other *Fusarium* metabolites (beauvericin, equisetin, aurofusarin) and *Alternaria* spp. toxins (sterigmatocystin, anthrachinone). As a consequence of the study cited above, the presence of the banned substance zeranol in urine of cattle was not attributable to illegal use, but to the natural occurrence of ZEN and α-ZEL in grasses.

## Case presentation

### Case description and diagnostic steps

Severe reproductive disorders occurred on a conventional farrowing farm with 200 sows and in-house gilt breeding (closed system). The farm was located in the valley of the Rhine in the southern country side neighboring Frankfurt am Main as the closest large city. Gestating sows were kept in groups of 30 sows (with 2.53 m^2^ per sow) with partially slatted floors, no bedding material and permanent access to a feeding station. Lactating sows were housed in farrowing crates. Before problems arose, a high productivity with 31 live born piglets and 28.5 weaned piglets per sow per year had been recorded, with average piglet losses of 8%.

**Fig. 1:**
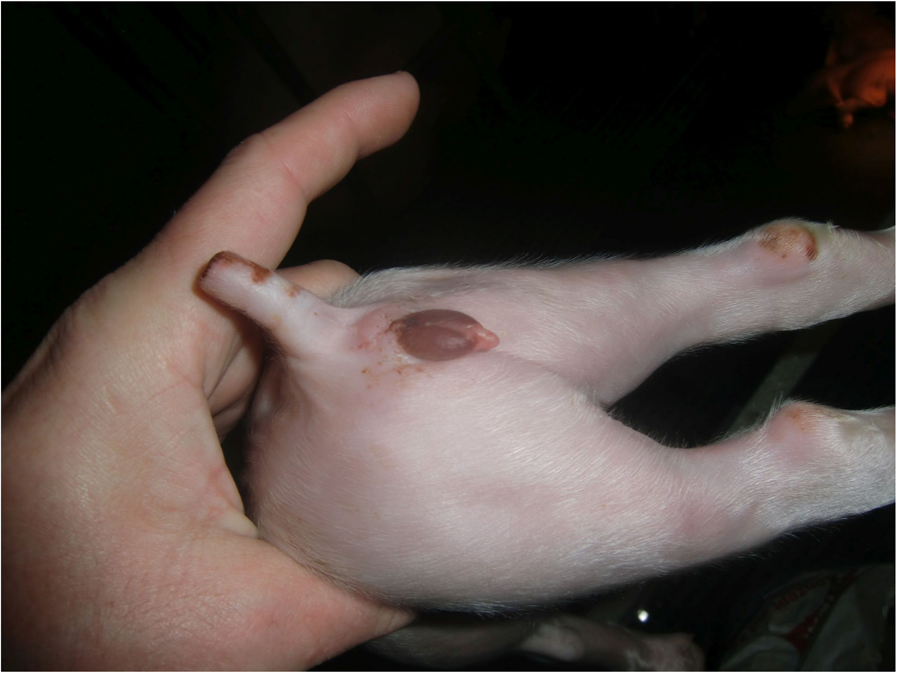
Two-day old piglet with a swollen vulva

The farmer complained about swollen external genitals of his piglets (Fig. 1) and higher percentages of weak live-born piglets with splay legs. Within two months, piglet losses during lactation increased up to 30% with estimated 21 weaned piglets per sow per year. Because the sows showed no fever or inappetence, and the symptoms found in piglets were characteristic for hyperestrogenism, the veterinarian had the suspicion of ZEN contamination of feed.

**Fig. 2:**
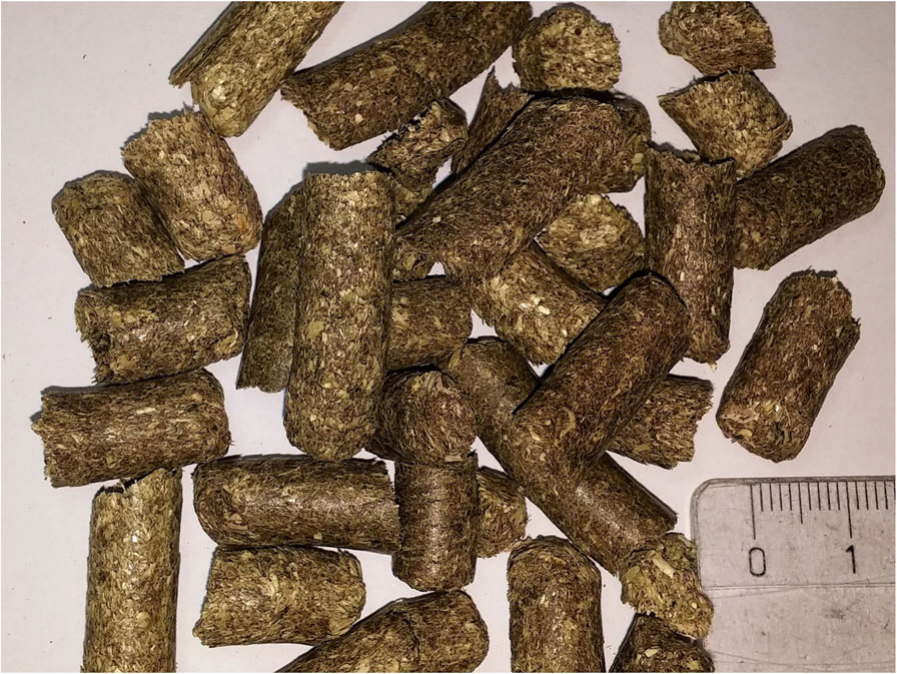
Hay pellets used in the diets (20–30 mm in length, 8 mm in diameter)

Feed composition is shown in Table 1. The farmer fed the sows two times a day with conventional diets containing crude fibre at ~ 7% in the diet for pregnant and 4.7% in the diet of lactating sows. The main source of crude fibre was hay in its first growth in the middle to the end of full bloom with an estimated protein content of 9%. The hay had an aromatic odor and had been harvested after three days of field-drying. In this period of dry weather conditions, the swath was turned daily, spread once (day 1), and was finally windrowed and baled (evening day 3). Hay pellets (Fig. 2) were produced by the feed mill for storage and ground prior to mixing with all other feed components. Routinely, three tons of feed for pregnant sows contained 210 kg of ground hay pellets.

**Table 1:** Feed composition in 1 kg (88%TS)

Feeding stuff	Pregnant sows	Lactating sows
Barley	42.5%	29%
Wheat	16.9%	41%
Corn		
Oat	20%	2%
Soy bean meal	10%	17%
Soy bean oil	1%	3%
Calcium carbonate	0.1%	
Minerals	2.5%	3%
Hay	7%	5%
Feed component		
Energy (ME)	12.2 MJ	13.4 MJ
Protein	138 g	161 g
Lysine	6.2 g	7.5 g
Methionin	2.2 g	2.4 g
Methionin+Cystein	5.2 g	5.6 g
Threonine	3.9 g	4.6 g
Tryptophan	1.5 g	1.8 g
Crude fibre	72 g	47 g
Fat	35 g	48 g
Starch	399 g	405 g
Calcium (Ca)	6.8 g	7.4 g
Phosphorous (P)	3.2 g	3.4 g
Ca/P	2.14	2.15
Lysine/ME	0.51	0.56
Lysine/100 g protein	4.46 g	4.66

Since the only change in feed batches in the very last months prior to the occurrence of clinical signs concerned the addition of ground hay pellets to the diet, the farmer had the suspicion of mycotoxin contamination of hay.

Feed sampling was performed by collecting subsamples (300 g) from five random lot locations. After combining and mixing these subsamples (1500 g), aliquots were subjected to different analyses. In a first step, selected feed components (barley, wheat, oat and hay pellets) were sent for mycotoxin detection by enzyme-linked immunosorbent assay (ELISA). While ZEN could not be quantified in barley, wheat and oat samples (< 20 μg/kg), analysis of hay pellets resulted in detected concentrations of > 500 μg/kg ZEN. Since mycotoxin occurrence in hay and associated risks are only rarely described in literature, verification of ELISA results by a validated liquid chromatography – tandem mass spectrometry (LC-MS/MS) method was performed. In addition, a plant component containing phytoestrogens was hypothesized as a potential cause for the clinical signs. This plant component would have not been discriminated by ELISA. Botanic analysis of hay samples for plant species determination was performed at the Institute for Animal Nutrition and Functional Plant Compounds, University of Veterinary Medicine Vienna, Austria. Hay pellets were softened in water and soluted for 60 min. Plant particles were distributed onto a slide and examined by 10× magnification for specific botanic structures.

In a next step, 1000 g hay pellets were sent in the framework of BIOMIN Holding GmbH’s mycotoxin survey program (Spectrum 380®) to the Center for Analytical Chemistry, University of Natural Resources and Life Sciences, Vienna, for extended mycotoxin analysis.

For quantitative analysis of mycotoxins, 5 g of milled sample were extracted using 40 mL extraction solvent (acetonitrile/water/acetic acid 79/20/1) followed by a 1 + 1 dilution using acetonitrile/water/acetic acid 20/79/1. Thereafter, 5 μL of the extracts were directly injected into the LC-MS/MS system. Screening of fungal metabolites was performed as described by Malachová et al. [25], but the method has in the meantime been expanded to cover 650 metabolites (manuscript in preparation). The accuracy of the method is verified on a continuous basis by regular participation in proficiency testing schemes including samples of complex animal feed [25, 26]. Results were corrected for apparent recoveries that have been determined by spiking experiments.

## Results

The botanic inspection of soluted hay pellets revealed the presence of grass components only, no herbs or other potential phytoestrogen producers were found.

The LC-MS/MS analysis of hay pellets resulted in 52 detectable fungal metabolites. Concentrations of the most relevant mycotoxins are given in Table 2. The sum value of ZEN and its modified forms reached 1040 μg/kg. Surprisingly, the levels of the modified mycotoxin ZEN-14-S (530 μg/kg) exceeded even those of its parent toxin ZEN (479 μg/kg), while concentrations of α-ZEL and β-ZEL were comparably low. DON, aflatoxin B_1_, fumonisins or ochratoxin A were not found. Detected concentrations of the trichothecenes nivalenol and T-2 toxin as well as of the *Alternaria* toxins alternariol and alternariolmethoylether were moderate, ranging from 6 to 79 μg/kg. The only toxic plant metabolite found was lotaustralin, a cyanogenic glycoside, with a concentration of 4130 μg/kg.

**Table 2:** Concentrations (μg/kg) of most relevant mycotoxins in analyzed hay pellet sample

Mycotoxin Group	Mycotoxin	Concentration (μg/kg)
Mycotoxins with regulated maximum/guidance levels^a^	Aflatoxin B_1_	< LOD^b^
	Sum of ergot alkaloids	27.4
	Deoxynivalenol	< LOD
	Sum of fumonisin B_1_ and fumonisin B_2_	< LOD
	Ochratoxin A	< LOD
	Zearalenone	479
Zearalenone derivatives	α-zearalenol	11.7
	β-zearalenol	16.9
	Zearalenone-glucoside	< LOD
	Zearalenone-sulfate	530
Other trichothecenes	3-Acetyl-Deoxynivalenol	< LOD
	15-Acetyl-Deoxynivalenol	< LOD
	Nivalenol	91.3
	T-2 toxin	78.9
	HT-2 toxin	< LOD
Alternaria toxins	Alternariol	47.8
	Alternariolmethylether	6.3

^a^According to regulations/recommendations set by the European Commission for livestock feeds [13, 72]

^b^LOD, limit of detection; exact values can be retrieved from [25, 26]

Considering the ELISA-based results for barley, wheat and oat (ZEN not quantifiable) and the inclusion rate of hay pellets, estimated levels of ZEN and its metabolites in the final diet for gestating and lactating sows were 72.6 μg/kg and 51.9 μg/kg, respectively.

One month after hay pellets had been removed from the diet and replaced by other fibre sources, no further clinical signs were observed in the newborn piglets.

## Discussion and conclusion

The botanic inspection did not provide indications concerning a potential source for the observed hyperestrogenism in suckling piglets, and ZEN could not be quantified in barley, wheat or oat. Thus, focus was laid on the results of LC-MS/MS analysis of the hay pellets. Regarding plant metabolites, elevated levels of lotaustralin were detected. In principle, hay contains not only grass, but also other plant species, e.g. some belonging to the family *Fabaceae*. Herbs were not detected by the botanic inspection, which is not sensitive enough to identify smaller plant particles in ground substrates. *Fabacea* and more than hundred other plant families frequently produce cyanogenic glucosides as botanic defense substances. The toxic substance hydrocyanic acid is derived from the cyanogenic glucoside lotaustralin by enzymatic activation in a relationship 1:10. In case of the analyzed hay sample, this would result in approximately 0.4 μg hydrocyanic acid per kg feed, which is far below those doses having toxic effects [27].

Thus, based on performed analyses, the presence of ZEN and its modified metabolites in hay samples remained as the only potential cause for the observed signs of hyperestrogenism in piglets. Clinical observations, linking vulvovaginitis in piglets to a ZEN exposure of the sow, were reported already in 1980 [28]. In general, piglets can be exposed to ZEN in utero [29] or by ingestion of the sow’s milk. The total ZEN intake of piglets via milk seems to be comparably low [30], even when considering potential release of ZEN from back fat of the sow [29]. Furthermore, a major metabolite found in sows´ milk is glucuronidated α-ZEL [30], which elicits only very low estrogenic activity [31]. Yet, bacterial β-glucuronidases might be capable of regenerating the aglycon in the intestinal tract of piglets, thus allowing α-ZEL to enter the enterohepatic circulation [32]. Although in utero exposure to ZEN is currently regarded as the primary source for ZEN-induced hyperestrogenism in newborn piglets [30], further research on this topic is definitely necessary to elucidate the role of the different exposure routes in more detail.

Literature data on dietary ZEN concentrations affecting pig health vary depending on factors such as exposure duration, administration type and age of animal. In general, severe effects on reproduction parameters of sexually mature gilts are observed at higher concentrations than reproductive symptoms in prepubertal female pigs [33]. Early field reports on the *perinatal estrogen syndrome* lack information on dietary ZEN concentrations and experimentally reproduced the observed effects only at unrealistically high concentrations of 40,000 μg/kg ZEN [28, 30]. After exposing gilts to up to 22,000 μg/kg ZEN, Kordic et al. [6] observed a clear negative impact on reproduction performance, e.g. increased number of dead-born piglets and occurrence of abortus, whereas vulvovaginitis in newly born female piglets was not evident. In a more recent case study on ZEN toxicosis in Serbian swine farms, observations included increased rates of rebreeding, infertility and anestrus in sows as well as dead born piglets and a large number of farrowed piglets with vulvovaginitis. Dietary ZEN concentrations as low as 720 μg/kg were monitored in feed for pregnant sows [34]. In the present case, levels ZEN and its modified forms reached 1030 μg/kg in analyzed hay pellet samples. As one feed component (soybean meal) was not tested, the concentrations of ZEN and its modified forms in the final diets could only be estimated. Based on the inclusion ratios of hay pellets, values of 72.6 μg/kg and 51.9 μg/kg total ZEN were calculated for the diets of gestating and lactating sows, respectively. Those levels are markedly below the levels reported by Prodanov-Radulović et al. [34] and the EU recommendations [13]. Under field conditions, factors such as immune status, stress exposure, general health condition, age or metabolism capacity can markedly influence the severity of mycotoxin-induced effects in individual animals. In the described case, long-term intoxication must be assumed due to the feeding of contaminated hay pellets for five months (July to November) and the and the toxicokinetics of ZEN [2]. The disappearance of clinical signs in newborn piglets one month after removal of hay pellets further support the assumption that ZEN contamination of the fibre source caused the hyperestrogenism. As such, we were neither able to confirm nor to completely exclude ZEN as definite source for observed disorders in the present case. This clearly highlights the need for reliable diagnostic tools, which allow veterinarians to deal with suspected cases of ZEN mycotoxicosis in a more conclusive manner.

So far, diagnosis of ZEN mycotoxicosis is mainly based on the observation of clinical signs and feed analysis. Yet, ZEN, as with many other mycotoxins, might exhibit adverse health effects in livestock before clinical signs become evident [35–37]. Analysis of feed samples, on the other hand, is accompanied by certain challenges. First, representative feed samples need to be collected, taking into account the non-homogeneous distribution of certain mycotoxins within a lot [38] as well as time point of sampling, as feed lots might have exchanged between the time point of sampling and the initial onset of mycotoxin-induced effects [34]. Also, the present case demonstrates that mycotoxin analysis should not be restricted to cereals and cereal byproducts, but rather include fibre sources and enrichement materials. Extensive surveys on the mycotoxin contamination of straw or hay are scarce. Concerning hay, 5–70% of samples were found positive for ZEN in Germany, Ireand, Canada and Ontario [39–41]. In those studies, maximum ZEN concentrations exceeded 1000 μg/kg ZEN only in one alfalfa-timothy mixed hay sample [41]. Yet, incidences of *Fusarium* spp. infection and ZEN levels in grasses vary depending on factors such as sampling site, variety, weather conditions, harvesting time and year [42]. Adequate hay making procedures and storage conditions are essential to limit subsequent mycotoxin production [43].

Next, one has to consider the occurrence of modified mycotoxins in feed. In the present case study, hay samples were not only contaminated by ZEN, but also contained significant amounts of ZEN-14-S. Risk assessment for this modified mycotoxin is currently ongoing. Although ZEN-14-S shows a clearly reduced estrogenic activity at the receptor level [44, 45], liberation of ZEN during mammalian digestion was indicated by in vitro experiments [46]. Only in 2017, the first report on the fate of ZEN-14-S in pigs became available, demonstrating that this modified mycotoxin was indeed hydrolysed in vivo [47]. Since a significant proportion of ZEN and other still-unknown metabolites were formed, the authors concluded that ZEN-14-S contributed to the total ZEN burden of pigs and should be considered in regulations. The same applies to other modified forms of ZEN, such as glucoside conjugates [42]. Due to the poor or unknown cross-reactivity of antibodies used in immunochemical methods [48, 49], chromatographic methods are currently preferable for the accurate determination of modified mycotoxins in feed [50] and are therefore recommended for proper assessment of total ZEN exposure of farm animals.

Furthermore, contamination of feed samples with multiple mycotoxins needs be considered, which can result in additive, synergistic or antagonistic effects in animals [51]. Although mycotoxin co-occurrence in feed is a rather common phenomenon with single samples containing up to 68 different metabolites [3], mycotoxin interactions are poorly investigated. Synergistic effects might be more pronounced at low mycotoxin concentrations [52], but results vary depending on tested mycotoxin combination, investigated parameter, animal species or exposure duration [51, 53]. Several fungal metabolites were detected in hay, but none of those exceeded existing maximum/guidance levels for feed or was present in otherwise considerable concentrations. Yet, the occurrence of *Alternaria* toxins alternariol and alternariolmethylether might be noteworthy. Vejdovszky et al. [54] recently demonstrated synergistic estrogenic effects of alternariol and ZEN in vitro, and also alternariolmethylether seems to elicit estrogenic effects [55]. However, further research is necessary to confirm those results in vivo and to obtain indications on toxin ranges inducing synergistic effects in swine. Unfortunately, neither the final diet nor other feed components were subjected to a multi-mycotoxin analysis. Thus, the full spectrum of sow’s mycotoxin exposure remains unknown, which is a clear limitation of our case study.

In contrast to feed analysis, measurement of specific ZEN biomarkers in biological matrices of swine, such as urine, feces, bile or tissue, would allow diagnosis of mycotoxicosis on more individual level. By doing so, variations in metabolism and toxin intake between individual pigs could be accounted for. According to Baldwin et al. [56], two types of biomarkers are used in mycotoxin research: exposure-based biomarkers and mechanism-based biomarkers. While the first approach describes the measurement of a mycotoxin itself and/or its metabolites, mechanism-based biomarkers refer to a specific biological response (e.g. increase/decrease in proteins or cellular metabolites) that can be linked to mycotoxin intake [56]. Concerning ZEN, major efforts have been made during the last years to establish an exposure-based biomarker in pigs. Under controlled conditions, numerous studies have investigated effects of increasing toxin concentrations on levels of ZEN and/or its metabolites in blood, urine and bile, but also in feces, cerebrospinal fluid, liver or back fat (e.g. [29, 57–62]). Usually, highest concentrations of ZEN and its metabolites are recovered in bile and urine [57, 61, 62]. In addition, better correlation coefficients (ingested ZEN versus recovered ZEN) were obtained for ZEN residues in those two matrices compared to blood [57, 61, 62], which can be partly attributed to very low blood levels of this mycotoxin. In support, a recent field study failed to correlate ZEN levels in sow feed to those in plasma as toxin concentrations in plasma were under the limit of quantification in the majority of samples [63]. Once a steady state is reached, total ZEN levels in urine are less prone to intra-day variations than those of DON [58]. This is easily explained by major differences in the kinetics of these toxins, with ZEN undergoing enterohepatic recirculation and having a comparably higher elimination half-life [64, 65]. On the other hand, this implies that ZEN can be detected in bile even at marginal dietary contamination levels, that are of no toxicological concern [61], or theoretically also in the complete absence of ZEN in feed [65].

Although all those studies performed under controlled conditions contributed significantly to our knowledge on ZEN and its toxicokinetics, exposure-based biomarkers can still not be recommended for the prediction of ZEN intake under field conditions. As nicely depicted by Dänicke and Winkler [65], comparable concentrations of ZEN in bile, blood or urine can derive from a broad range of ZEN exposure levels. Most of all, ZEN residues do not provide indications on the severity of adverse health effects induced by ZEN, as demonstrated e.g. by the absence of a correlation between the total ZEN levels in bile and the uterus weight [65]. Consequently, establishment of reference values for toxicologically relevant ZEN concentrations in biological matrices is currently not feasible.

Unfortunately, the situation does not look too promising for mechanism-based biomarkers either. Besides its estrogenic potency, ZEN has also been described to exhibit genotoxic and immunomodulatory effects [2]. Alteration of various biological measures after ZEN exposure has been shown in vivo, including hematological and biochemical parameters (e.g. [61, 66]) as well as mRNA expression (e.g. [67–69]). In recent years, focus has also been laid on impact of ZEN on the intestine [70]. However, reproducibility of induced effects as well as specificity of investigated parameters is limited. To the best of our knowledge, no clear dose-response relationship between the increase/decrease of biological measures in blood, urine or bile and ZEN intake has been established. As stated by Gajęcka et al. [66], low levels of dietary ZEN might induce entirely different metabolic changes than high concentrations, which hinders the establishment of a mechanism-based biomarker. In this respect, the evolving omics-technologies might be a suitable tool to unravel the toxicodynamics of ZEN [71] and thus contribute to the identification of novel and applicable biomarkers.

To conclude, the present field case serves as good example for the various challenges associated with the diagnosis of ZEN mycotoxicosis in pigs. Currently, feed analysis provides the only analytical tool to assist diagnosis of ZEN-induced disorders on farms. However, potential limitations concerning the sampling procedure and covered mycotoxin spectrum have to be considered in interpretation of results. As shown in this study, enrichment materials and other fibre sources can contain significant amounts of (modified) mycotoxins and should be therefore implemented in feed analysis.

## Abbreviations

Ca: Calcium
DON: Deoxynivalenol
ELISA: Enzyme-linked immunosorbent assay
HPLC: High performance liquid chromatography
LC-MS/MS: Liquid chromatography - tandem mass spectrometry
LOD: Limit of detection
P: Phosphorous
ZEN: Zearalenone
ZEN-14-S: Zearalenone-14-sulfate
